# Identifying children with excess malaria episodes after adjusting for variation in exposure: identification from a longitudinal study using statistical count models

**DOI:** 10.1186/s12916-015-0422-4

**Published:** 2015-08-06

**Authors:** Francis Maina Ndungu, Kevin Marsh, Gregory Fegan, Juliana Wambua, George Nyangweso, Edna Ogada, Tabitha Mwangi, Chris Nyundo, Alex Macharia, Sophie Uyoga, Thomas N Williams, Philip Bejon

**Affiliations:** KEMRI-Wellcome Trust Research Programme, Kilifi, Kenya; Department of Medicine, Imperial College, London, UK; Centre for Clinical Vaccinology and Tropical Medicine, University of Oxford, Oxford, UK

**Keywords:** Distribution, Malaria, *Plasmodium falciparum*, Poisson model, Simulation, Zero inflated binomial model

## Abstract

**Background:**

The distribution of *Plasmodium falciparum* clinical malaria episodes is over-dispersed among children in endemic areas, with more children experiencing multiple clinical episodes than would be expected based on a Poisson distribution. There is consistent evidence for micro-epidemiological variation in exposure to *P. falciparum*. The aim of the current study was to identify children with excess malaria episodes after controlling for malaria exposure.

**Methods:**

We selected the model that best fit the data out of the models examined and included the following covariates: age, a weighted local prevalence of infection as an index of exposure, and calendar time to predict episodes of malaria on active surveillance malaria data from 2,463 children of under 15 years of age followed for between 5 and 15 years each. Using parameters from the zero-inflated negative binomial model which best fitted our data, we ran 100 simulations of the model based on our population to determine the variation that might be seen due to chance.

**Results:**

We identified 212 out of 2,463 children who had a number of clinical episodes above the 95^th^ percentile of the simulations run from the model, hereafter referred to as “excess malaria (EM)”. We then identified exposure-matched controls with “average numbers of malaria” episodes, and found that the EM group had higher parasite densities when asymptomatically infected or during clinical malaria, and were less likely to be of haemoglobin AS genotype.

**Conclusions:**

Of the models tested, the negative zero-inflated negative binomial distribution with exposure, calendar year, and age acting as independent predictors, fitted the distribution of clinical malaria the best. Despite accounting for these factors, a group of children suffer excess malaria episodes beyond those predicted by the model. An epidemiological framework for identifying these children will allow us to study factors that may explain excess malaria episodes.

**Electronic supplementary material:**

The online version of this article (doi:10.1186/s12916-015-0422-4) contains supplementary material, which is available to authorized users.

## Background

Malaria remains a major public health problem with approximately 60 % of the world’s population at risk of infection [1]. The current antimalarial drugs and insecticide-dependent control methods are at risk from the emergence of resistant parasites and mosquitoes, respectively. Additional control methods, such as preventative vaccines, are therefore required. Vaccine development could be guided by understanding why some individuals are more susceptible to clinical malaria than others. Through continuous exposure to malaria parasites, children acquire immunity to clinical malaria as they grow older [2]. However, the distribution of clinical malaria is highly heterogeneous, even among children of similar ages as demonstrated in Kenya [3, 4] and in Senegal, where the numbers of clinical episodes ranged from zero to 40 per child over a 5-year period of surveillance in the same village [5]. In another study, a subgroup of children suffered malaria attacks every 4 to 6 weeks over many years for unexplained reasons [6, 7].

Like many other infectious diseases, malaria shows heterogeneity of transmission [8], which may explain this variation in the distribution of clinical malaria. There is renewed interest in examining the fine-scale geographical variation in exposure to infected mosquito bites that may account for this [9–11]. However, there are indications that host susceptibility to clinical malaria may also vary [12]. For instance, a number of genetic polymorphisms have been associated with innate resistance to *Plasmodium falciparum* malaria. These include the sickle cell trait (haemoglobin AS genotype; HbAS), thalasaemias, and blood group (reviewed in Gong et al. [13]).

Previously, we described a group of children who suffer multiple episodes of clinical malaria in Kenya [3]. In the current study, we confirm and extend these findings by analysing a large data set comprising two different cohorts including 2,463 children over 15 years of follow-up, and by additional adjusting for micro-epidemiological variations in exposure [14]. This allowed us to identify and characterise a group of children who experienced more episodes of clinical malaria than would be expected based on their exposure.

## Methods

### Ethics

Approval for human participation in these cohort studies was given by Kenya Medical Research Institute Ethics Research Committee, and research was conducted according to the principles of the Declaration of Helsinki, which included the administration of informed consenting in the participant’s local language.

### Cohorts

The active weekly clinical surveillance-platform for the collection of the data analysed is based on two cohorts of children living in rural subsistence farming villages at the coast of Kenya [15, 16], Junju and Ngerenya, where 1,235 and 1,259 children had been followed by December 2013 and December 2002 from the inception of the cohorts, respectively. Junju and Ngerenya were founded in 2005 and 1998, respectively, and have been under continuous weekly active surveillance for malaria ever since. Children are recruited at birth in study homesteads, and exit follow-up at 15 years of age. Junju is under moderate malaria transmission intensity with *P. falciparum* parasite prevalence from cross-sectional surveys at 30 % during January to May [17, 18], while Ngerenya is in an area in which malaria transmission has fallen to very low levels since 2004 [19], such that older children were historically exposed but younger ones have not been. For that reason, we excluded all the follow-up data from Ngerenya collected from 2002 onwards due to paucity of malaria episodes. In this region, *P. falciparum* is primarily transmitted during two periods of increased precipitation each year: May through July, and October through December [17, 18].

Asymptomatic infections were assessed from blood smears collected during annual cross-sectional surveys conducted during the dry season.

### Malaria diagnosis and treatment

Children were visited in their homes by field workers that lived in their midst to determine malaria-associated fevers. An episode of malaria is defined as axillary temperature ≥37.5°C associated with >2,500 *P. falciparum* parasites per microliter of blood. In addition, where children were positive for malaria with the rapid diagnostic test, thick and thin blood smears were prepared and subsequently stained with 10 % Giemsa and examined at ×1,000 magnification for asexual *P. falciparum* parasites. In total, 100 fields were examined before slides could be considered negative. Malaria in this area has been treated with co-artemether since 2005. Previously, it was amodiaquine, introduced in 2003 after the failure of sulfadoxine/pyrimethamine due to increases in resistant *P. falciparum* parasites.

### Determination of asymptomatic *P. falciparum* infections

Asymptomatic *P. falciparum* infections were assessed by microscopy at annual cross-sectional surveys at the end of the dry season where the level of malaria transmission is very low.

### Analysis

We analysed data collected over a period of 16 years (1998–2013) from 2,463 children from Kilifi county aged between zero and 15 years that were actively followed for the determination of frequencies of clinical malaria, constituting a total of 573,264 field observations and 11,371 child-years of observation. After determining the number of malaria episodes for each child, we fitted various count models including Poisson, negative binomial, and zero-inflated negative binomial models using child-years as the unit of observation (i.e. to allow for varying age and calendar time in order to account for trends in transmission intensity in the study area with time and “exposure index (EI)” during the time of follow-up). EI is a marker of the level of an individual’s exposure to malaria, and was calculated as the distance-weighted prevalence of clinical malaria within 1 km radius of the child’s residence as previously described [14, 20, 21].

We determined the best model for our data by comparing the Akaike information criterion (AIC) and we determined that zero-inflated negative binomial was a better fit than negative binomial using the Voung test. For the non-linear fits, we used the multivariable fractional polynomial routine from Stata, and quoted the *P* values based on comparisons between the linear and non-linear effects. We simulated the model 100 times in order to determine the random distribution of excess malaria (EM), using Poisson functions, a gamma distribution with the parameters returned by the model fit and a chance of zero-exposure for a given year based on the zero-inflation parameter. We placed an arbitrary cut off at the 95^th^ percentile of excess malaria (observed minus expected episodes). We did not use observed divided by expected since a number of children had zero expected episodes. For parasitaemia and temperature, we compared the children with “excess (EM)” and “average (AM)” numbers of clinical malaria episodes by Student’s *t*-test.

## Results

**Fig. 1:**
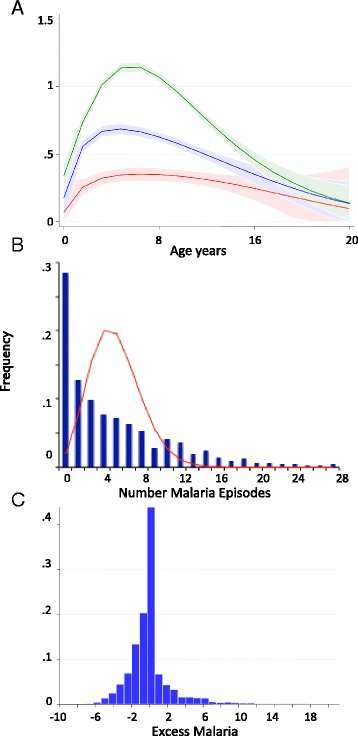
Distribution of malaria episodes. Panel **a** shows the distribution of episodes by age-blocks. Children are stratified by the amount of exposure to parasites in their environment into three tertiles; green line, highest exposure index; blue line, medium exposure index; and red line, lowest exposure index. Panel **b** shows an overlay of the expected Poisson over the observed distribution of numbers of clinical malaria episodes. Panel **c** is the distribution of excess malaria (observed minus expected) determined after 100 simulations of the zero-inflated binomial distribution of the numbers of clinical episodes

As expected from previous studies [2], the number of clinical episodes varied with increasing age showing an initial increase and subsequent decrease in risk, consistent with initially increased mosquito biting rates as the child grows and loss of protective maternal antibodies, and subsequent acquired natural immunity to clinical malaria (Fig. 1a). This increase and decline was most evident among the children living with the highest exposure to *P. falciparum* parasites in the microenvironment (i.e. with the highest exposure index), followed by those in the medium, and then the lowest of the three exposure index tertiles.

In order to demonstrate the degree of over dispersion in an unadjusted analysis, we first restricted analysis to 5 years of surveillance for each individual. The mean number of clinical malaria episodes for children that had accumulated within the 5 years of surveillance was 0.6 per child per year. A Poisson distribution based around this mean would predict that very few children would have a sum of more than eight episodes of febrile malaria (i.e. <1 %) from at least 5 years of follow-up. However, this was not the case and over 16 % of the children had more than eight episodes (Fig. 1b). The Poisson distribution is therefore a poor fit for the data.

**Fig. 2:**
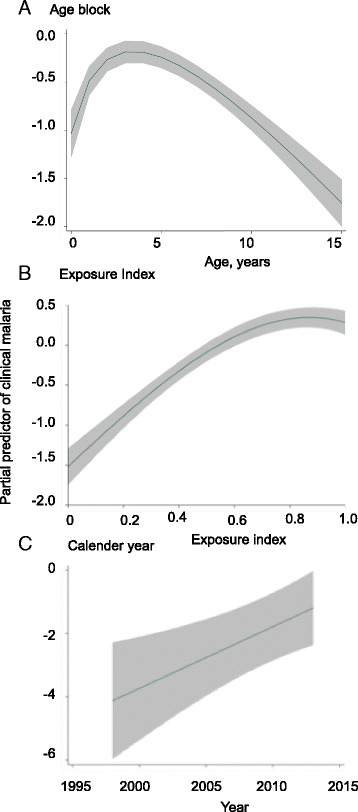
Fractional polynomial plots showing relationships between age, exposure index, and calendar year with numbers of clinical malaria. **a** Age (in years) was broken down into several blocks. **b** Exposure index, an estimate for the local prevalence of malaria for individual children. **c** Calendar years during which the respective clinical data were collected

We then included the full data set with adjusting for time at risk for each individual in order to compare how well different statistical models fit the data. We compared Poisson, zero-inflated Poisson, negative binomial, and zero-inflated negative binomial models adjusting for measures of *P. falciparum* exposure in the microenvironment: exposure index, calendar year, and age. We included significant (i.e. *P* <0.05) non-linear effects using multiple fractional polynomials as described previously [22] and used child-years of observation as the unit of analysis in these models, and therefore clustered individual observations by child using the robust-sandwich estimator to account for linked observations. The zero-inflation models used exposure index as a logistic function to predict the risk of zero counts. The negative binomial model was a significantly better fit for the data than the Poisson model as judged by the AIC test (AIC = 16,811). Similarly, the zero-inflated binomial model was a significantly better fit than the negative binomial model when the two were compared by Voung test (i.e. Z = 3.17, *P* = 0.001). This is not surprising, considering that 47 % (1,167 of 2,463) of the children included in the model accumulated zero episodes and the distribution clearly did not fit a Poisson distribution. The relationships between age, EI, and calendar year with malaria, the building blocks of the model, are shown by plotting the partial predictors and residuals in Fig. 2. The final model with non-linear transformations is shown in Additional file 1.

Having determined the best fitting model out of those tested, we then ran 100 simulations applying the parameters returned from the model to the covariates observed in our population and selected children above the 95^th^ percentile for observed minus expected numbers of clinical cases as an EM group (Fig. 1c). We noted a positive skew with an excess of children with extreme positive observed–expected values. We restricted this analysis to children with 5 or more years of follow-up to reduce uncertainty. We then compared this EM group with a group of children matched for similar EI but who had only experienced an average number of clinical episodes (AM), defined as children with the expected number of cases of malaria or fewer, as determined by the zero-inflated negative binomial model. We aimed to match each EM child with the closest two AM controls, one with higher and one with lower EI. There were 212 out of 2,463 children that fulfilled our criteria for EM and we identified 319 exposure-matched AM children. The average time in follow-up and age at entry were similar between the AM and EM groups: 8.03 (confidence interval (95 % CI), 7.8–8.3) vs 7.70 (95 % CI, 7.6–8.2), *P* = 0.5, and 1.88 (95 % CI, 1.64–2.11) vs 1.75 (95 % CI, 1.55–1.95) years, *P* = 0.4, respectively. Exposure indices were 0.51 (95 % CI, 0.48–0.53) vs 0.49 (95 % CI, 0.47–0.51), *P* = 0.2, for EM and AM children, respectively. Insecticide-treated net usage was similar between the EM and AM children (*P* = 0.9, Fisher’s exact; 84 % vs 83 %, respectively).

**Fig. 3:**
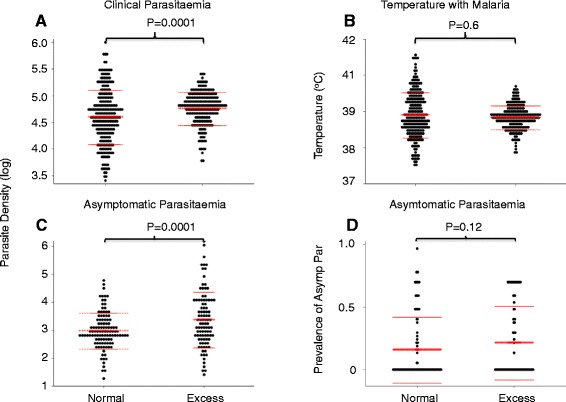
Differences in the levels of parasitaemia and axillary body temperature between excess malaria (EM) and age-matched average malaria (AM) controls. EM children were matched to AM children by EI, where both groups of children have been under active weekly surveillance for at least 5 years. Panels **a** and **b** compare the levels of parasitaemia and temperature during clinical malaria. Panel **c** compares the levels of asymptomatic parasitaemia during cross-section surveys done before malaria transmission. Panel **d** shows the prevalence of positive blood smears per individual children over several cross-sectional surveys

Compared to AM, the EM children had (1) a higher geometric mean parasitaemia density during asymptomatic infections determined from cross-sectional surveys of blood smears in the dry season when *P. falciparum* transmission is minimal [4,597 (95 % CI, 1447–7746) vs 43,891 (95 % CI, 8,500–79,282) parasites per μL], *P* = 0.0001; Fig. 3 panel b), (2) a higher geometric mean parasitaemia density during clinical malaria diagnosed from active weekly surveillance [80,230 (95 % CI, 67,718–92,742] vs 72,056 (95 % CI, 65,804–78,307) parasites per μL], *P* = 0.0001; Fig. 3 panel c), and (3) were less likely to be of the sickle cell (HbAS) trait genotype that is known to protect from malaria (Table 1). Although the proportion of children admitted to hospital with malaria was higher amongst EM than AM children, the differences were not significant (Table 2). There were no differences in the levels of axillary body temperatures during clinical malaria (Fig. 3b). Finally, there was no significant difference in the prevalence of asymptomatic *P. falciparum* positive blood smears at pre-transmission cross-sectional surveys (0.22 (CI, 0.16–0.28) vs 0.16 (CI, 0.12–0.21), *P* = 0.2, for EM and AM, respectively; Fig. 3d).

**Table 1:** Sickle cell trait protects against excess malaria

	Group		
Genotype	Normal	Excess	*P* value
AA	281 (77.8 %)	204 (96.7 %)	0.001
AS	78 (21.6 %)	7 (3.3 %)	0.001
SS	2 (0.6 %)	0	–
Total	361	211	

The numbers in brackets are percentages out of the total for the column. Fisher’s exact test was applied to test for differences

**Table 2:** Common causes for hospital admission in the cohort

Diagnosis	Group		
	Average malaria	Excess malaria	*P* value
Malaria	12 (13.7)	11 (19.7)	0.5
Febrile convulsions	4 (4.5)	4 (7.2)	0.7
Gastroenteritis	11 (12.5)	7 (12.5)	1
Lower respiratory tract infections	12 (13.6)	7 (12.4)	1
Urinary tract infections	5 (5.7)	2 (3.6)	0.7
Bronchiolitis	2 (2.3)	1 (1.8)	1
Epilepsy	1 (1.1)	1 (1.8)	1
Total	88	56	

The numbers in brackets are percentages out of the total for the column. There was no evidence for a statistically significant difference in the total numbers of admissions between the two groups, Fisher’s exact test *P* = 0.432. The groups were also compared by Fisher’s exact test

**Fig. 4:**
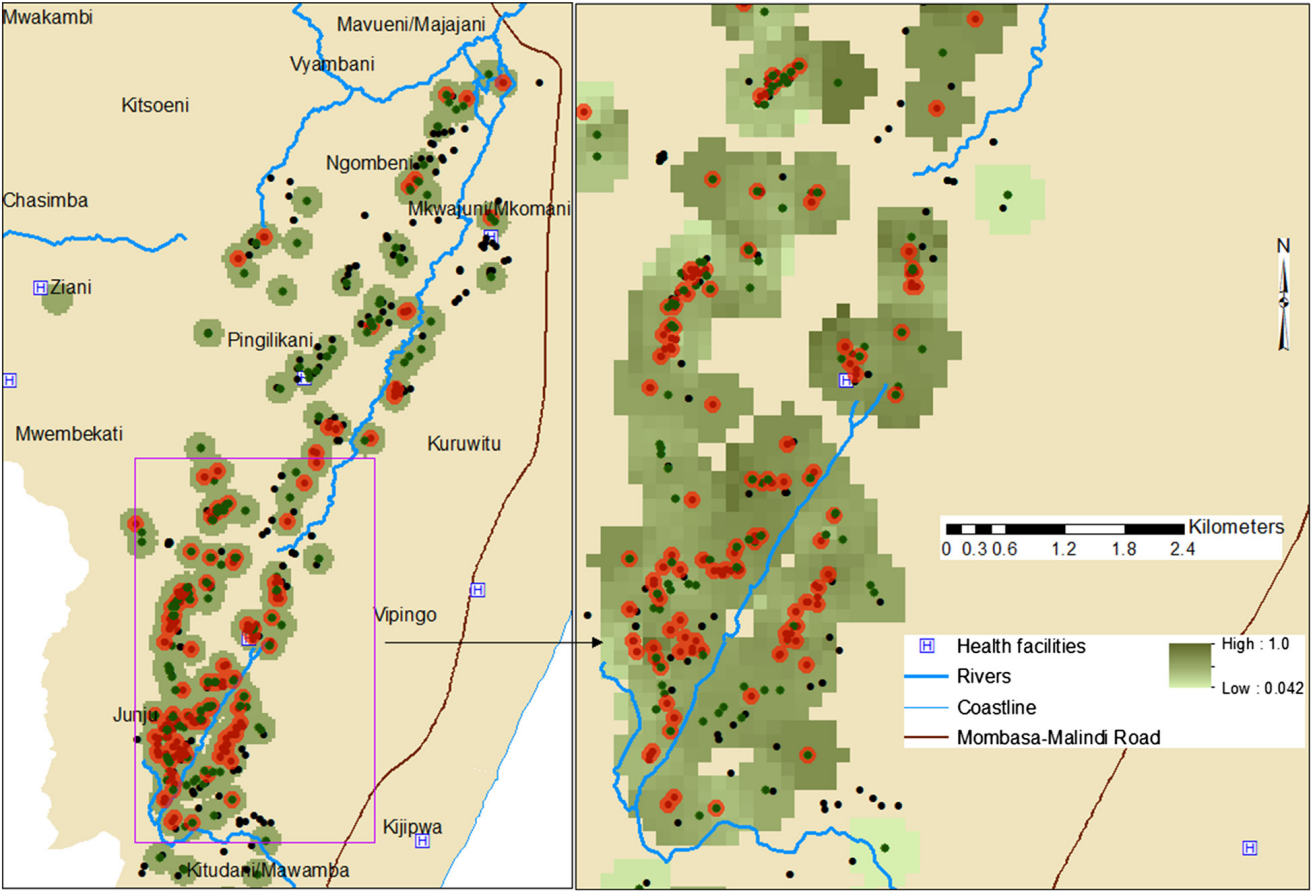
Geographical distribution of excess malaria (red dots) and average malaria (dark green dots) children in one of the study locations, Junju (2005–2013). The gradation from light green to dark green correlated with low to high exposure to malaria in the homesteads. The black dots mark the location of study homesteads

Both EM and the matched AM children were frequently found in the same geographical locations, further supporting the idea that having excess malaria is not always explained by environmental factors like increased exposure to malaria in the microenvironment (Fig. 4). For example, 79 out of 173 EM children in this study area (Junju) shared homesteads with at least one or more AM children (Fig. 4).

All these epidemiological markers point to EM as a group of children, who, over a sustained period of time, fail to acquire the ability to control parasite growth. Together, our analyses suggest that the factors responsible for increased susceptibility in the EM group are host dependent and not environmental, with sickle-cell trait being an obvious example tested.

We compared the outputs of classification of EM based on the zero-inflated binomial regression model and the output of a simple cut-off, varying the cut-off from >5 to >10 clinical episodes irrespective of exposure and other variables (Additional file 2). There was no cut-off that closely replicated the results of the model, the best compromise was a cut-off of >7 episodes, for which 27 children were classified as EM by the model but not by the cut-off, and 11 children were classified as EM by the cut-off but not by the model.

## Discussion

Previous studies demonstrated heterogeneous distributions of the numbers of accumulated malaria episodes for individuals, even amongst children of similar ages living in the same villages [3, 5, 23]. There is good evidence to show that variation in exposure to infected mosquito bites, which may in turn be influenced by the host factors, including behaviour, economics, attractiveness to mosquitoes, and other factors [4, 24, 25]. However, there is also evidence for variations in host susceptibility [12, 26]. Herein, we have used a zero-inflated negative binomial model controlling for exposure in the microenvironment to identify children with EM that may be attributed to individual factors. We found children with similar levels of exposure to *P. falciparum*, similar levels of bed net usage, and similar ages but with some having suffered excessive numbers of clinical episodes. We also found EM and AM children frequently living close to each other or even sharing the same homestead.

Furthermore, EM children had increased parasite densities upon asymptomatic infections and during clinical malaria compared to AM controls, suggesting that they have reduced immunity to control malaria infections. Furthermore, the increased parasite densities and the higher proportion of EM children admitted to hospital with a diagnosis of malaria point to the possibility that clinical malaria in these children would have been more severe than malaria in AM children if left untreated, or with delayed treatments. In interpreting this result we should note that because the children in these cohorts are under active weekly surveillance for malaria, they receive more timely treatment for malaria upon diagnosis than the general population. Hence, we assume that the risk of hospital admission due to malaria would be higher in the general population.

These findings are in agreement with our previous study which also described a group of children with increased susceptibility to clinical malaria in Kenya [3]. In the current study, we confirm and extend these findings by analysing a large data set comprising two different cohorts including 2,463 children over 15 years of follow-up, and by additional adjusting for micro-epidemiological variations in exposure.

A study in Senegal has also shown that asymptomatic parasite densities increased with age among a group of children with multiple episodes of malaria [27]. In contrast, asymptomatic parasite densities in the general population decreased with age, leading the authors to suggest that these children were failing to acquire anti-parasite immunity. Furthermore, in a separate study conducted in Kenya, pre-transmission asymptomatic infections in the absence of antibodies were found to be a risk factor for clinical disease in the ensuing malaria transmission [28].

In the current study, we carefully controlled for variations in exposure, including use of EI, to predict the risk of a zero count, representing a sub-population of children with no exposure during a year of follow-up [29]. However, we cannot capture all variation in exposure, which may vary even within a homestead. In support of a role for host factors, we found that HbAS is strongly protective against excess malaria. HbAS is well known to protect against clinical malaria [30] and therefore failure to identify HbAS as a significant factor distinguishing EM from AM children would have indicated that variation in exposure was still a dominating factor.

An additional finding was the increased risk of clinical malaria with calendar year between 2005 and 2013. This is consistent with wider trends in malaria transmission described on the Kenyan Coast [31], and could be explained by reductions in the level of immunity (and hence increasing numbers of susceptible individuals) following a prolonged period of reducing *P. falciparum* transmission. We included the possibility of interactions between calendar time and site, and between calendar time and age, and discarded these because they were not significant.

## Conclusion

We might expect that children of the same age and living with the same amount of exposure to malaria or the in the same geographical location would be similarly susceptible to clinical malaria. However, this study indicates that there may be a skewed distribution, with some children experiencing excessive numbers of episodes, perhaps indicating that these children fail to acquire natural immunity to malaria. However, there may be other inter-individual variations in exposure or behaviour patterns between children or their parents that are poorly accounted for by the model and could also explain the skewed distribution of children with excess episodes. Further studies of immunological parameters will be required to provide further evidence for the hypothesis that host immunity contributes to the variation observed. Furthermore, such studies will also reveal biomarkers for identifying such children with EM, which could in theory be used for targeting interventions to a small group of children that are responsible for a disproportionate amount of malaria-driven morbidity and mortality. Moreover, if these children represent the 20 % predicted to be responsible for the majority of malaria transmission [25], targeting them with interventions and control methods would be more cost effective at reducing the overall transmission than targeting whole populations.

## Additional files

Additional file 1:
**Final zero-inflated model.** (PDF 43 kb) Additional file 2:
**A comparison of the numbers of excess malaria as classified by simple cut-offs or by the negative binomial model.** (PDF 33 kb)

## Abbreviations

AIC: Akaike information criterion
AM: Average malaria
EI: Exposure index
EM: Excess malaria
HbAS: Haemoglobin AS
*P. falciparum*: *Plasmodium falciparum*
