# Supplementary material for: Identifying children with excess malaria episodes after adjusting for variation in exposure: identification from a longitudinal study using statistical count models
Source: BMC Med. 2015 Aug 6;13:183. doi: 10.1186/s12916-015-0422-4 (PMC4527301; doi:10.1186/s12916-015-0422-4)
Supplement: Additional file 1: — Final zero-inflated model. (PDF 43 kb) [file 12916_2015_422_MOESM1_ESM.pdf]

Final Zero Inflated Model model

Covariates (generated from mfp nbr model)	Incident Rate Ratio	P	95% Confidence Interval
(Exposure Index+2.30e-17)) – 0.43	14.75	0.0001	8.31 – 26.71
((Exposure Index+2.30e-17))^{3} – 0.08)	0.27	0.0001	0.19 - 0.41
(Age +1)/10)$^{0.5}$-0.77	102.12	0.0001	36.12 – 288.66
(age+1)/10)$^{0.5}$*ln(age_block+1)/10)+0.40	0.02	0.0001	0.01 - 0.04
Calender year-2006.871542	1.03	0.0001	1.02 – 1.04
ln(Age*Exposure Index +1.73e-18)/10)+1.48	0.89	0.0001	0.87 – 0.92
ln(Age*Exposure Index +1.73e-18)/10)^{2}-2.20	0.99	0.0001	0.996 - 0.998
Inflate			
Exposure Index	-2.40	0.005	-4.07 – -0.74
Lnalpha	-0.46	0.032	-0.88 - -0.04
Alpha	0.62		0.41 – 0.96

Footnote: Age is in years, Exposure index is a weighted prevalence scale ranging from 0 to 1.
