# Supplementary material for: Identifying children with excess malaria episodes after adjusting for variation in exposure: identification from a longitudinal study using statistical count models
Source: BMC Med. 2015 Aug 6;13:183. doi: 10.1186/s12916-015-0422-4 (PMC4527301; doi:10.1186/s12916-015-0422-4)
Supplement: Additional file 2: — A comparison of the numbers of excess malaria as classified by simple cut-offs or by the negative binomial model. (PDF 33 kb) [file 12916_2015_422_MOESM2_ESM.pdf]

A comparison of the numbers of EM as classified by simple cut-offs or by the negative binomial model

Arbitrary cut off	>5	>6	>7	>8	>9	>10
Number of children with episodes less than the simple threshold, but classified as EM by the model	3	10	27	36	63	95
Number of children with episodes more than the simple threshold, but not classified as EM by the model	64	29	11	1	0	0
This table illustrates how simple/arbitrary cut-offs of excess malaria compare with the zero inflated binomial model.						
